# Numerical analysis of a multiproduct biorefinery on a chip: Exploiting acoustic waves to process the microalgae *Tisochrysis lutea*

**DOI:** 10.1016/j.ultsonch.2025.107280

**Published:** 2025-02-16

**Authors:** Jacques R.N. Kieffer, Hakan Kandemir, Lars Stegemüller, Isa Hiemstra, Michel H.M. Eppink, Rene H. Wijffels, Iulian Z. Boboescu

**Affiliations:** a Bioprocess Engineering, AlgaePARC, Wageningen University & Research, Wageningen, the Netherlands; b Department of Electrical Engineering and Automation, Aalto University, Helsinki, Finland; c Faculty of Biosciences and Aquaculture, Nord University, Bodø, Norway

**Keywords:** Microalgae, Downstream Processing, Biorefinery, Acoustophoresis, Microfluidics, Acoustic Contrast Factor

## Abstract

Microalgae can provide a more sustainable alternative to traditional food systems which are dominated by terrestrial crops. The main economic challenges, however, relate to the downstream processing of microalgae and the valorization of their side streams. The present work explores the scientific principles and data required to develop an integrated biorefinery-on-a-chip, which replaces many of the common downstream processing unit operations by employing acoustic fields. The acoustic parameters of *Tisochrysis lutea* microalgal cells and their cell components are determined using the neutrally buoyant state method. Culture conditions which result in a high carbohydrate or high protein to lipid ratio led to a higher acoustic contrast factor than culture conditions favoring a high composition of lipids. The collected acoustic data is used as input in a numerical model which studies the harvesting of microalgal cells and the fractionation of microalgal cell components. High separation levels are achieved based on the size and composition of microalgal cells and the type of cell component. Subsequent studies are envisioned to determine the practical feasibility of applying these concepts and even scaling them out. Nevertheless, this study represents a steppingstone towards a novel, label-free approach to processing microalgal cells of different biomass compositions.

## Graphical abstract

**Figure:**
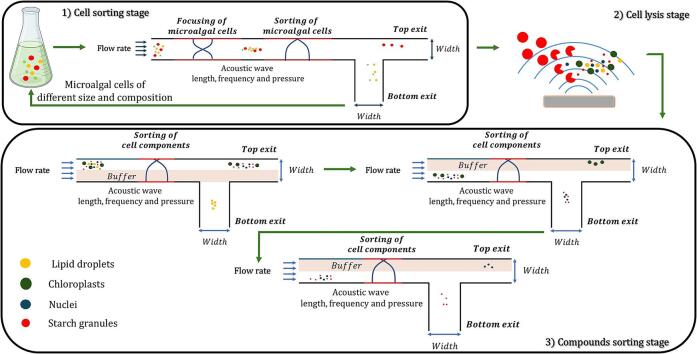

## 1 Introduction

Current food systems and chemical manufacturing processes further exacerbate the rapid degradation of the planetary boundaries [1]. Microbial biotechnology could alleviate some of these issues by providing alternative food and feed ingredients with high functionality as well as platform molecules for packaging and manufacturing [2]. These processes do not require arable land, fertilizers or pesticides, and can utilize various side streams or other inexpensive substrates. Moreover, the conversion efficiencies of microorganisms such as bacteria, yeast, fungi and microalgae are several orders of magnitude higher than terrestrial plants and animals [3]. Microalgae are considered excellent candidates for these processes as they can directly convert CO_2_ into lipids, proteins, carbohydrates, pigments and secondary metabolites using light energy [4]. However, processing these microalgal cells and retrieving the desired products is often one of the biggest bottlenecks preventing these approaches from becoming economically feasible [5].

Most common processing steps currently employed in microalgal biotechnology are biomass harvesting to remove part of the water, cell disruption to release their target components and product fractionation to purify these compounds prior to their use [6]. Biomass harvesting is often performed through passive or enhanced settling using polymer flocculants, or active approaches such as filtration and centrifugation [7]. The drawbacks of these approaches are linked to extensive processing times, as in the case of settling, expensive flocculants, membrane fouling as well as harsh and energy intensive, as in the case of centrifugation. The subsequent cell disruption step is commonly performed through harsh mechanical approaches such as high-pressure homogenization or bead milling [8]. In some cases, when processing species with no– or thin- cell wall, multiple cycles of freeze-thawing are employed [9]. However, these technologies are very harsh and generate heat which risks denaturing the structure of the released compounds, thereby negatively impacting their activity. Furthermore, freeze-thawing is limited in its use-cases and is energy intensive as well as time consuming [10]. Lastly, the extraction and purification of the released compounds is mostly performed via solvent extraction. Using organic solvents however is harsh and hazardous to human health and the environment, as well as energy- and chemically- intensive. Their application leads to safety risks, high costs, loss of compound functionality and loss of secondary products [11]. In some cases, chemically driven precipitation or chromatography approaches are being utilized [12]. However, these either require the extensive use of acids and bases, or very expensive chromatography columns, unsuitable for bulk compounds.

Due to the aforementioned limitations of current processing technologies, the focus is shifting towards alternative approaches in pursuit of milder, tunable and multiproduct solutions. For instance, enzyme assisted cell disruption has been evaluated to decrease the stiffness of the cell wall and thus the required mechanical treatment intensities. This led to reduced shear forces, lower temperatures and thus better product quality in terms of its functionality [13]. However, due to the high diversity of cell wall structures and cost of these enzymes, these approaches are relatively limited. Ionic liquids on the other hand have been explored to both weaken the cell wall structures, as well as provide a tunable solvent for specific compounds [14]. These properties, together with the multitude of different potential combinations and low volatility resulted in increased interest regarding these novel solvents. However, most of them remain toxic, are not bio-based and make the back-extraction of the recovered product extremely difficult. To address this, recent years have seen a shift towards using natural eutectic solvents as a bio-based and less-toxic alternative [15]. Challenges remain within these eutectic systems as well, being characterized by high viscosity and low volatility. In an attempt to increase even more the efficiency and sustainability of these processes, the integration of external fields has been investigated in recent years [16]. These show to enhance the efficiency of certain downstream unit operations such as biomass disruption and extraction of various compounds, while reducing the amount of chemicals required [6]. Among these technologies, acoustic fields seem a very promising avenue due to their multitude of phenomena such as acoustic cavitation leading to generation of shear forces, targeted heat and free radicals which can be employed in sonochemistry applications [17]. Moreover, acoustic fields provide the ability to manipulate and interact with individual cells and cellular structures through acoustic stationary waves generating primary and secondary acoustic radiation forces and streaming effects [18].

Acoustic fields are generated through vibrations which give rise to propagating pressure waves within a medium. To induce vibrations it is common to use a piezoelectric crystal, henceforth referred to as transducer, which creates mechanical motion when subjected to an AC current (reverse piezoelectric effect) [19]. When pressure waves from coherent (e.g. equal frequency) vibration sources interact, they interfere constructively and destructively. This can be achieved either by two opposing transducers or a transducer and a highly acoustically-reflective surface. These interferences generate a stationary wave pattern with precisely defined loci of high pressure (i.e. pressure anti-node) and low pressure (i.e. pressure node) [20]. Alternatively, when two opposing pressure waves with slightly different frequencies interact, a quasi-stationary wave pattern with moving nodes and anti-nodes are generated, where the moving speed depends on the frequency difference between sources [21]. A particle which enters this stationary or quasi-stationary pressure field can be subjected to a range of phenomena, among which a nonzero time-averaged force termed acoustic radiation force (ARF). Depending on the particle acoustic contrast factor (ACF) the ARF can accelerate the particle either towards a pressure node or a pressure antinode. The intensity of the ARF is proportional to the frequency and amplitude of the acoustic field, the particle radius, position and ACF [22]. The latter is determined by the difference in specific gravity and compressibility between the medium and the particle. A positive ACF leads to a force on the particle toward the pressure node and a negative ACF leads to a force toward the pressure anti-node. By strategically generating pressure (anti)-nodes within a continuous system the ARF can be used to precisely manipulate the position of particles [23]. This phenomenon is termed acoustophoresis.

As there are multiple methods of generating a desired pressure field in liquids, acoustophoresis can be achieved on different platforms. For example, a microchannel can be excited by bulk acoustic waves (BAW), via an external piezoelectric transducer, such that the acoustic pressure field in the channel is utilized for particle trapping or continuous separation. It is thus imperative to utilize adequate fabrication materials for these systems to minimize energy losses as the acoustic waves are transmitted through the chip itself. It is also very important to consider the acoustic impedance ratio of the materials in such devices, as the impedance ratio plays a crucial role in minimizing losses due to impedance mismatch as well as being essential for generating stationary waves [24], [25], [26], [27], [28]. Another method is to use surface acoustic waves (SAW) that leak into the microfluidic channel [29], [30], [31], [32], such that the stationary wave in the channel is created by the surface waves on the substrate. SAW are generated by interdigitated transducers (IDT), whose architecture, and in particular the spacing between the interdigitated transducers, is matched to the excitation frequency. While the chip material of choice may be narrower than BAW resonators, more versatile and well controlled acoustic fields can be generated by SAW resonators. When higher frequencies are utilized in both of these systems, secondary hydrodynamic contributions can be observed, known as acoustic streaming [29]. These disturbances can contribute to the ARF from a fraction of its component, to orders of magnitude larger. In the latter case, this becomes the dominant driving force manifested within the system. Recent efforts however demonstrated the potential of minimizing the acoustic streaming effects in microchannels when these are not desired [30], [31]. Both BAW- and SAW-operated systems have been studied in ever-improving numerical models in literature [32], [33], [34], [35]. Alternative to the traditional ways of creating the stationary waves via BAW or SAW, the resonances in the whole chip can also be utilized to employ acoustophoresis in microchannels, while the whole chip including the transducer is resonating. In addition, the external transducer can be replaced by a thin film transducer within the resonator without sacrificing the performance [32], [36], [37]. Finally, an alternative to stationary fields are dynamic acoustic fields. These are employed across various platforms, generating well-controlled quasi-stationary waves for particle manipulation [21], [38], [39].

Acoustic waves show a high potential for processing microalgae due to the ability to fine-tune them, leading to label-free manipulation and capacity to interact mildly with non-charged and non-magnetic molecules [40]. Acoustofluidic systems have been researched across many different biological and non-biological particle systems such as polystyrene beads [41], [42], microorganisms [43], [44], [45], [46] and blood cells [47], [48], [49]. Apart from within separation devices, acoustic waves may be used to induce disruption of the microalgal cell membrane and cell wall by cavitation or provoking mechanical resonance [50], [51]. This could either enhance current disruption and extraction methods, or even replace them entirely [17]. Research on the acoustic processing of microalgae predominantly focused on the cell lysis aspects using various acoustic frequencies, intensities and processing times. Some work was also directed on the acoustic harvesting of these cells within microfluidic devices, with notable results [52]. However, these focused mostly on separating larger cells (20–30 µm) of certain species from smaller particles or cells of another species. Findings regarding their ACF differences have been reported as well [40]. However, in most biotechnological applications single species are used to accumulate specific compounds such as lipids, proteins or carbohydrates. In addition, these species tend to be much smaller (2–7 µm). Thus, it is unclear if acoustic radiation forces alone could be fine-tuned sufficiently to separate cells of the same species based only on slight differences among their size and biomass composition. Moreover, to the best of our knowledge, the acoustic separation of released cellular structures such as chloroplasts, lipid droplets and starch granules has not yet been investigated.

The hypothesis is that acoustic fields can not only be used to mildly disrupt microalgal cells but also drive the other processing steps required in a multiproduct biorefinery while accounting for changing microalgal growth stages and culture conditions. The aim is hence to study the selective harvesting of different microalgal cells, for example microalgae with a certain diameter or with accumulated lipid droplets or starch granules, and fractionate their cell components after their release through a mild cell disruption. Since the study of this system takes place within a microfluidic environment, this concept is termed biorefinery-on-a-chip. To envision such a process, understanding the correlations between changing acoustic properties of the microalgal cells under different conditions and growth stages and their susceptibility to acoustic manipulation needs to be studied. Additionally, revealing the acoustic properties of cellular structures such as lipid droplets, starch granules, chloroplasts, pyrenoids and nuclei, may enable the development of novel acoustic-based extraction and separation approaches. The primary challenge of implementing acoustic separation unit operations within industrial processes is linked to energy efficiency. Energy costs are generally considered high for acoustic separation devices, however, this is expected to improve by designing more efficient electrical driving circuits [53]. Furthermore, the chip building material and architecture can significantly reduce energy costs by improving acoustic impedance matching and attenuation characteristics. Scaling out these biorefinery-on-a-chip devices through parallelization, i.e. driving multiple microfluidic channels in parallel, or designing larger fluidic systems could make them competitive in terms of separation efficiency, energy efficiency and throughput for current industrial applications [54].

The presented work focuses on identifying the acoustic properties of *Tisochrysis lutea* (T-iso) cells and some of its components as well as how these change as a response to variations in biomass composition. T-iso is a marine flagellate microalgal species currently used as a natural supplement in aquaculture feed. This species has recently received increasing interest due to the high concentration of fucoxanthin and long-chain omega-3 polyunsaturated fatty acid [55]. The data is subsequently used to perform numerical studies and optimizations on an acoustic microfluidic chip model to specifically deviate microalgal cells of a certain composition and even cell components such as chloroplasts, lipid droplets and starch granules towards different outlets of this chip. The acoustic cell lysis is not included in this work as it was thoroughly discussed by the authors in a previous study [17]. To the best of our knowledge, this is the first time a multiproduct acoustic biorefinery-on-a-chip is explored. The gained insights could pave the way for the development of a comprehensive solution integrating all of the main processing steps such as cell harvesting, lysis and product fractionation on a single continuous microfluidic system, with ramifications in high-throughput product development and even larger scale biomass processing.

## 2 Materials and methods

### 2.1 Microalgal cultures

T-iso microalgae provided by NECTON, S.A. (Olhão, Portugal), were cultivated throughout 25 days in 250 mL shake flasks with approximately 100 mL of culture. The cultures were grown under three different conditions (Table 1). The first condition, representing the control, was cultivated under optimum conditions at 25 °C, 90 rpm, 0.2 % (v/v) CO_2_, 16/8 day/night cycle and 90–130 µmol m^−2^ s^−1^ incident light. Natural seawater collected from the North Sea (The Netherlands) was used as culture medium, enriched with 3.04 g L^-1^ NaNO_3_ (Sigma-Aldrich), 2 mL/L NutriBloom (Phytobloom, NECTON) micronutrient solution and 4.76 g L^-1^ HEPES buffer (Sigma-Aldrich). The pH of the medium was adjusted to 8.0 using NaOH and autoclaved for 20 min at 121 ⁰C. The second culture condition, the temperature-stressed one, was cultivated using the same media composition as the control, but at 20 °C, 85 rpm, 2.5 % (v/v) CO_2_, 16/8 day/night cycle and approximately 150 µmol m^−2^ s^−1^ incident light. Lastly, the third T-iso culture condition represents the nitrogen-starved one. Cultivation took place under identical conditions as the control but without the addition of NaNO_3_.

**Table 1:** **The varying parameters used throughout the cultivation of the three different T-iso cultures.** The other growth variables such as mixing speed, light intensity and regime, as well as CO_2_ concentration were constant for all three cultures.

Growth condition	Temperature	Addition of nitrogen
Control	25 °C	Yes
Temperature-stressed	20 °C	Yes
Nitrogen-starved	25 °C	No

### 2.2 Biomass analysis

#### 2.2.1 Biomass pretreatment

T-iso microalgal biomass was regularly harvested to measure biomass production and composition. Pre-weighed glass vials were used to determine the biomass concentration prior to analysis. 2–3 mL of T-iso culture was added to pre-dried glass vials and dried overnight in the oven at 100 °C after 3 series of washing with 3 mL 0.5 M ammonium formate to remove the salts and pelletizing T-iso by centrifugation at 1200 *g* for 5 min. The difference between the dried glass vial with and without sample, divided by the volume of the sample resulted in the biomass concentration in g L^-1^. All measurements were performed in triplicate. Additionally, the washed and pelletized biomass was freeze-dried for approximately 13 h and stored at −20 °C. For further biomass analysis the dry pellets were ground to a powder, using a spatula.

#### 2.2.2 Carbohydrates

Approximately 1 mg of freeze-dried T-iso biomass was weighed and lysed by adding 0.5 mL 2.5 M HCl and incubating for 3 h in a water bath at 100 °C. The tubes were vortexed every hour to ensure homogenous lysis. Once the samples cooled to room temperature, 0.5 mL of NaOH were added prior to centrifuging for 10 min at 1200 g. For the analysis, 50 µL of the supernatant were transferred into a glass tube and diluted with 450 µL MilliQ water. The calibration curve was setup from 500 µL standard glucose solution in a concentration range of 0 to 0.1 g L^-1^ in 5 steps. 500 µL of a 5 % phenol solution were added to the standards and samples without mixing. Subsequently, 2.5 mL 95–98 % sulfuric acid was dispensed in glass vials and incubated for 10 min at room temperature followed by 30 min in a water bath at 35 °C. The absorbance was measured at 483 nm, with the 0 g L^-1^ glucose sample serving as the blank. The concentration of carbohydrates was determined through the calibration curve and biomass weight. All measurements were performed in triplicates.

#### 2.2.3 Fatty acids

5 to 10 mg of the biomass powder was mixed with 1 mL chloroform:methanol mixture (2:2.5 v/v) inside bead beating tubes and placed in the homogenizer (Precellys 24, Bertin Instruments, France) [17]. The samples underwent 8 cycles of 60 s each with 120 s pause at 800 g. The lysed cells were subsequently transferred to 10 mL glass tubes by washing three times with chloroform:methanol mixture (2:2.5 v/v) to minimize cell loss. The tubes were vortexed and sonicated for 10 min at 80 kHz using an ultrasonic water bath (Sonation, Germany). This step was repeated after adding 2.5 mL of Tris buffer (50 mM Tris + 1 M NaCl at pH 7.5). Subsequently, the samples were centrifuged for 5 min (800 g, 15 °C), resulting in a phase separation, followed by the recovery and evaporation under nitrogen gas of the chloroform phase containing the lipid extracts.

The subsequent methylation was performed by adding 3  mL methanol containing 5 % (v/v) H_2_SO_4_, followed by vortex and incubating the samples for 1 h at 100 °C. Subsequently, 3 mL MiliQ water and 1 mL hexane were added and vortexed for 15 min in a test tube rotator. After centrifuging for 5 min at 800 *g* and 15 °C, the top hexane phase was transferred to gas chromatography (GC) vials and analyzed using a Shimadzu GC, Japan. The inlet temperature was set to 250 °C with hydrogen as carrier gas at a pressure of 81.7 kPa, a split ratio of 0.1:1, and a total flow rate of 20 mL min^−1^. The FID (flame ionization detector) was operated at a temperature of 270 °C with a hydrogen flow rate of 40 mL min^−1^, an air flow rate of 400 mL min^−1^ and a helium flow rate of 9.3 mL min^−1^. The injection volume was set to 1 µL per sample, including a pre- and post-wash of the injector with n-hexane. All measurements were performed in triplicates.

#### 2.2.4 Proteins

Approximately 3 mg of freeze-dried biomass was dissolved in 1 mL of 0.4 M NaOH and incubated for 30 min at 100 °C. The samples were centrifuged for 10 min at 1500 *g* and the supernatant was diluted twofold with 0.4 M NaOH. A concentration range of 0–1.4 mg mL^−1^ BSA in 5 steps was prepared from a 2 mg mL^−1^ BSA standard in 0.4 M NaOH by diluting with a 60 mM Tris, 2 % SDS lysis buffer (pH 9). The analysis was performed using a protein assay kit, Bio-Rad Dc in 96-well microplates. In brief, reagent A’ was prepared with 1 mL of reagent A and 20 µL of reagent S. First, 10 µL of each sample and standard was transferred into the microplates followed by the addition of 25 µL of reagent A’ and 200 µL of reagent B. The plates were covered with aluminum foil and incubated for 30 min. The absorbance at 750 nm was subsequently measured using an Infinite (Tecan, Switzerland) microplate reader. All measurements were performed in triplicates.

#### 2.2.5 Pigments

One mL of microalgal culture was centrifuged at 4400 rpm for 8 min at 4 °C. 5 mL of 100 % methanol was added to the pellet and treated for 5 min in the ultrasound bath at 80 KHz. Subsequently, the cell suspension was incubated at 0 °C for 15 min followed by centrifugation at 4400 rpm for 8 min. The extraction step was repeated until the pellet became completely white. The chlorophyll *A* and B as well as carotenoid content was spectrophotometrically measured at 470 nm, 652 nm and 665 nm in a quartz cuvette. The blank was performed using methanol. All measurements were performed in triplicates. Arnon’s equations were used to calculate the chlorophyll and carotenoid content [56]:

(1) $$\mathrm{Ch}l_{a}=\left(16.72\cdot A_{665.2}-9.16\cdot A_{652.4)}\right)\cdot dilutionfactor$$

(2) $$\mathrm{Ch}l_{b}=\left(34.09\cdot A_{652.4}-15,28\cdot A_{665.2}\right)\cdot dilutionfactor$$

(3) $$\mathrm{Ch}l_{\mathrm{tot}}=Chl_{a}+Chl_{b}$$

(4) $$\mathrm{Caro}t_{\mathrm{tot}}=\frac{\mathrm{dilutionfactor}\cdot 1000\cdot A_{470}-1.63\cdot Chl_{a}-104.96\cdot Chl_{b}}{221}$$

### 2.3 Acoustic properties of T-iso microalgae and their chloroplasts

The specific gravity of these cells as well as their compressibility (derived from the particle speed of sound) were determined indirectly using the neutrally buoyant state of intact T-iso cells and extracted chloroplasts [57]. In brief, Percoll solutions containing 5 ✕ GR Buffer, Isoascorbate buffer, PCBF solution and water were made at increasing concentrations. Fresh intact T-iso cells were diluted with sea water to an OD_750_ of 0.4 and 1 mL was transferred to a 1.5 mL Eppendorf tube and centrifuged at 4500 *g* for 5 min with the supernatant being subsequently discarded. The obtained pellet was resuspended and washed with 10 mL 0.5 M ammonium formate to remove salts. Subsequently, the cell pellets were gently resuspended in Percoll solutions of increased concentrations (from 5 % to 30 % in incremental steps of 5 %) and centrifuged at 4500 *g* for 5 min. The Percoll concentration in which the microalgal cells are still uniformly suspended and do not form a pellet nor float on the top of the tube, and thus in a neutrally buoyant state, was analyzed in triplicate using the DSA 5000 M density and velocity meter (Anton Paar, Austria).

Similarly, the acoustic properties of the T-iso chloroplasts were determined after a previous isolation step modified from [58]. T-iso microalgal cultures cultivated for 20 days under optimal conditions were diluted to an OD_750_ of 0.75 and washed as described above. First, the pellet was resuspended in 300 mM sucrose, 2 mM EDTA and 1 mM MgCl_2_ and centrifuged (3,000 g, 10 min, 4 °C). The pellet was resuspended in 100 mM sucrose, 2 mM EDTA and 1 mM MgCl_2_ and put on ice for 20 min before being transferred to 2 mL Lysing Matrix C tubes (MP Biomedicals^TM^) containing 1.4 mm ceramic spheres and bead beaten for two cycles of 2,500 rpm, for 10 s per cycle (Precellys 24 Homogenizer, Bertin Instruments). Subsequently, the disrupted samples were transferred to Falcon tubes and centrifuged for 10 min at 800 g, 4 °C. The supernatant, containing the chloroplasts, was set aside and the pellet was resuspended in the 100 mM sucrose solution and bead-beaten again using the same settings to increase the chloroplast yield. The supernatant obtained from the two cell disruption rounds was then centrifuged for 10 min at 1,000 g to remove intact cells, followed by a second centrifugation at 4,500 g for 10 min to pelletize the chloroplasts. This was gently resuspended in 8 mL of isolation buffer as previously reported [59]. For the Percoll density gradient layering, 10 % (v/v), 30 % (v/v) and 50 % (v/v) were prepared. The layers were accumulated from bottom to top with decreasing density (50 %, 30 %, 10 %, respectively). Per density layer, 10 mL was used. 5 mL of sample was gently loaded on top of the three layers and centrifuged for 30 min at 8,000 g, 4 °C. After centrifugation, the formed band at the 50–30 % interface was collected and resuspended in Percoll solutions of increased concentrations, as described in the previous paragraph, to determine the acoustic properties of the isolated chloroplasts.

The results are used to indirectly determine the ACF of the T-iso intact cells as well as the extracted chloroplasts in the neutrally buoyant state through Equation (5).

(5) $$\phi =\frac{1}{3}\left(\frac{5\rho_{p}-2\rho_{m}}{2\rho_{p}+\rho_{m}}-\frac{\beta_{p}}{\beta_{m}}\right)$$

where ϕ is the ACF, ρ the specific gravity and β the compressibility of the particles (p) and the medium (m) they are suspended in [22]. To approximate the growth media commonly used for microalgae, the values for the specific gravity and compressibility were taken from water at room temperature.

### 2.4 Numerical analysis

#### 2.4.1 Theoretical underpinnings

A particle suspended in a medium experiences acoustic radiation force when it is perturbed by an acoustic wave. Consequently, the acoustic radiation force acting on the particle can be expressed by the surface integral of the pressure, where the integration surface is the surface of the particle. Such a calculation is cumbersome, since the particle may have an arbitrary shape and the surface of the particle moves because of the acoustic radiation force. Alternatively, the force can be calculated by transforming the integral surface to a fixed regular (e.g. spherical) surface surrounding the particle (Equation (6)).

(6) $$F=-\langle \underset{S_{p}\left(t\right)}{\int \;\;\;\;\;\int \;◯}p\;{\vec{n}}_{p}\;dS_{p}\rangle \;=-\langle \underset{\Omega }{\int \;\;\;\;\;\int \;◯}\left(p\;+\rho \vec{v}\vec{v}\right)\vec{n}\;d\Omega \rangle$$

In the above expression, $S_{p}\left(t\right)$ and ${\vec{n}}_{p}$ denote the surface and normal vector of the particle surface, and Ω is the fixed surface surrounding the particle. $p=p_{0}+p_{1}+p_{2}$, $\vec{v}={\vec{v}}_{0}+{\vec{v}}_{1}+{\vec{v}}_{2}$ and $\rho =\rho_{0}+\rho_{1}+\rho_{2}$ are the pressure, particle velocity and density, respectively. Quantities with subscript 0 are equilibrium quantities, and quantities with subscript 1 are the first-order disturbances caused by the acoustic wave. The second order quantities, $p_{2}$ and ${\vec{v}}_{2}$, result in the acoustic radiation pressure and acoustic streaming, respectively. The brackets 〈〉 represent the time-average over one period; periods of acoustic waves in especially MHz range are so short that the force is not exactly resolved. Furthermore, since the first order quantities have harmonic time dependence, it is only the second order terms resulting in the acoustic radiation force and streaming, and the second order terms can be expressed by using only the first order terms [22].

By using scattering theory, the incident and scattered fields on a spherical particle can be expressed in terms of incoming fields. Denoting the incoming pressure and velocity fields $p_{\mathrm{in}}$ and ${\vec{v}}_{\mathrm{in}}$ respectively, the acoustic radiation force on a spherical particle is given as

(7) $$F=-\nabla \frac{4}{3}\pi r^{3}\left(f_{1}\frac{1}{2}\beta_{m}\langle p_{\mathrm{in}}^{2}\rangle -f_{2}\frac{3}{4}\rho_{m}\langle v_{\mathrm{in}}^{2}\rangle \right)$$

where $f_{1}=1-\frac{\beta_{p}}{\beta_{m}}$ is the monopole scattering coefficient and $f_{2}=\frac{2\left(\frac{\rho_{p}}{\rho_{m}}-1\right)}{2\left(\frac{\rho_{p}}{\rho_{m}}\right)+1}$ is the dipole scattering coefficient. The combination of these coefficients results in the acoustic contrast factor. When the incoming wave is a stationary wave, such as $p_{\mathrm{in}}\left(y,t\right)=P_{0}\cos\left(ky\right)cos\left(\omega t\right)$, the acoustic radiation force on a spherical particle can be further simplified. There are also forces due to scattering from multiple particles, but provided that the particles are not in concentrated solutions, the interaction forces can be ignored [29]. A spherical particle in a stationary acoustic wave field is thus subjected to an acoustic radiation force [22]:

(8) $$F_{y}\left(y,t\right)=4\pi kr^{3}E_{\mathrm{AC}}\phi \sin\left(2ky-∊t\right)$$

here, $k=2\pi f/c_{0}$ (m^−1^) is the wavenumber determined by the frequency of excitation and the speed of sound in the host medium, r (m) is the particle radius $E_{\mathrm{AC}}=\frac{P_{0}^{2}}{4\rho_{0}c_{0}^{2}}$ (Jm^−3^) is the acoustic energy density, ϕ (−) is the ACF of the particle, y (m) is the particle position relative to the pressure node, and ∊=2πΔf (s^−1^) is the angular frequency difference between two opposing sources (with average frequency of f) in case of a dynamic acoustic field. If ∊≠0, the stationary wave pattern and in turn the force landscape moves with v=∊/2k. In case of ∊=0, the time dependency of the force vanishes, and the expression is identical to the force expression in a stationary wave field.

In addition to the acoustic radiation force, particles experience drag force due to the relative motion between the particles and the surrounding medium, given by the Stokes’ drag force expression (Equation (9)).

(9) $$F_{y}^{\mathrm{Drag}}=6\pi \mu r\dot{y}$$

where μ is the viscosity of the surrounding medium and $\dot{y}$ is the relative velocity of the particle with respect to the surrounding medium.

Typically, the acoustic radiation force and drag force counteract each other, and their different scaling factors are exploited in acoustofluidic systems. For a spherical particle in a stationary wave field, the time required to travel between initial position $y_{0}$ and a final position $y_{f}$ is calculated as (Equation (10)):

(10) $$t=\frac{3\mu }{4\Phi kr^{2}E_{\mathrm{AC}}}\ln\left(\frac{\tan\left(ky_{f}\right)}{\tan\left(ky_{0}\right)}\right)$$

Utilizing this information while designing the channel, it is possible to put significant distance between different particles such that they will be guided to different exits, i.e. separated.

As an alternative, two-frequency type dynamic acoustic fields can be used to put distance between particles instead of stationary acoustic fields. Assuming the dynamic wave field moves in a perpendicular direction to the flow field, the following dimensionless number defines whether the particle is captured or not.

(11) $$K=\frac{2kr^{2}E_{\mathrm{AC}}\Phi }{3\mu rv}$$

In the above expression, if a particle has K≥1, it is captured and moved by the dynamic acoustic field. Dynamic acoustic fields require multiple pressure nodes in a channel to be effective, hence they are suitable for wider, multi-wavelength resonators, offering higher throughput due to larger dimensions of the channel. Theory for such fields and other practical considerations can be found in work by [21].

Finally, the ACF offers another method for separation. Particles with different ACFs are attracted to different regions in the field. This method is very suitable to separate lipid components, as they have a negative ACF, from other cell components such as starch granules, nuclei and chloroplasts that all have positive ACFs. Depending on the excitation frequency, the size of the cell components may become comparable to the viscous boundary layer thickness. In this case, viscosity-corrected ACFs should be used [22].

While the second order pressure field leads to acoustic radiation force, the second order velocity field leads to acoustic streaming. This is especially true when targeting sub-micrometer particles requiring higher frequencies to deflect, which also leads to the formation of these streaming fields. In these specific cases, the streaming forces can indeed become stronger than the primary acoustic radiation force, leading to undesired particle behavior. However, by using proper channel design, materials and/or buffers, the acoustic streaming can be minimized or even completely suppressed [60], [61]. Thus, considering these potential mitigations and in order to maintain focus on the primary acoustic radiation force, as well as the relatively low frequencies explored and generally larger particle size, we neglected the streaming effect throughout the paper, while not forgetting to emphasize the importance of considering it during the design of an acoustofluidic chip.

In addition to the acoustic forces, the particles are experiencing drag force due to the fluid flow, eventually making the particles follow the fluid flow. The drag force in microfluidic flows can be modelled as Stokes drag force. Considering the flow profile, the drag force in x-direction is given as

(12) $$F_{x}^{\mathrm{Drag}}=6\pi \mu r\dot{x}\left(y\right)$$

Finally, in real-world applications, it is essential to obtain the acoustic fields as close to the theoretical modelling as possible. In the actual device, the acoustic field may be distorted, which is adversely affecting the particle concentration. Nevertheless, the continuous flow smoothens out the particle tracks neutralizing the effect of distortions [62]. As discussed in earlier sections, there have been many studies on obtaining minimal-distortion fields as well as acoustic streaming suppression. Among the considered platforms, SAW devices come up marginally ahead, for the amount of research on suppressing streaming, as well as the possibility to use stationary wave fields and dynamic acoustic fields on the same chip. Nevertheless, the equations and considerations employed throughout this paper are equally applicable to both BAW and SAW.

#### 2.4.2 COMSOL® simulations

To explore the feasibility and opportunities of harvesting microalgal cells with varying properties and fractionating their internal structures, numerical analyses were carried out by using a simple-geometry acoustofluidic model in the FEM software COMSOL® Multiphysics 6.1. The acoustic parameters were retrieved from the experiments described in Section 2.3. and set as particle parameters. In order to calculate the particle trajectories, equation of motion for particles is solved in two dimensions.

(13) $$\left(\frac{4}{3}\pi r^{3}\right)\ddot{y}+6\pi \mu r\dot{y}+F_{y}\left(y,t\right)+F_{L}\left(x,y,t\right)=0$$

(14) $$\left(\frac{4}{3}\pi r^{3}\right)\ddot{x}+6\pi \mu r\dot{x}\left(y\right)=0$$

Pressure acoustics, frequency domain (acpr) physics is utilized to involve the acoustic fields and viscosity-corrected ACFs in the solver, laminar flow (spf) physics are used to solve for the fluid flow, and finally particle tracing for fluid flow (fpt) is used to solve the coupled differential equation of motion. For the solution of the flow field, the walls have Navier slip condition, the incoming flow is taken as fully developed, while the outlets are zero-pressure outlets. The acoustic fields are calculated by the velocity and impedance conditions of the walls, and for later simulations the field is used as background field in order to speed up simulations. Particles are released from designated inlets, and as the mixture is assumed to be not concentrate, the particle–particle interactions are ignored. The lift force on particles due to the velocity gradient is included by including the lift force node and included in the EOM as $F_{L}\left(x,y,t\right)$.

**Fig. 1:**
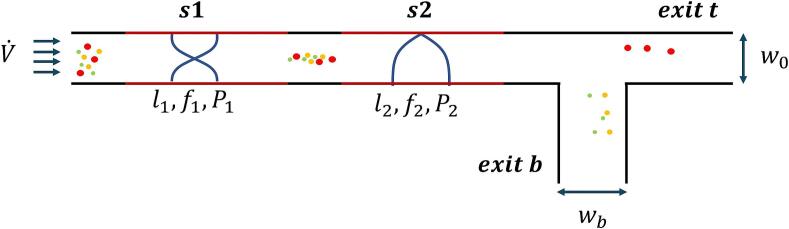
**Schematic of the acoustic microfluidic architecture used for harvesting T-iso.** Microalgal cells of different sizes and compositions, depicted by different colours, are first focused in the initial half-wavelength section, while the later quarter-wavelength separation section harvests the target microalgal cells (red). Each section is characterized by its length, the frequency and pressure of the acoustic field applied. The colours are used only to exemplify the variation of cell sizes and concentration within a heterogenous microalgal culture, and do not represent a specific type.

A common chip architecture enables the individual stages to be repeated and simplifies the optimization process (Fig. 1). For this simplistic bifurcation architecture, repetition of the individual stages is required if the goal is to fractionate multiple compounds, as each bifurcation (or stage) separates only one compound from the rest. The separation is based on the different time of flight of different particles, and since particles are entering randomly, a focusing section in the beginning is introduced to get the same time of flight for identical particles. Note that this is similar to having separate inlets for buffer liquid and concentrated particles, as done in [24].

In the diagram given in Fig. 1, $\dot{V}$ is the input flow rate and $l_{s}$, $f_{s}$, $P_{s}$ are the length, frequency of excitation and pressure in the focusing section (s1) and separation section (s2), respectively. $w_{0}$ is the width of the main channel that finishes in top exit t and determines $f_{1}$ and $f_{2}$. $f_{1}$ is chosen such that it will form a half-wavelength acoustic field within the main channel, with the node in the middle and thus focusing all particles with a positive ACF in the middle of the channel. $f_{2}$ is chosen such that a quarter-wavelength acoustic field is formed and a pressure node becomes apparent on one side of the wall of the main channel, thus deviating the positive ACF particle towards that wall. $w_{b}$ is the width of the perpendicular channel section that leads to exit b. To evaluate the harvesting and fractionation model parameters, the efficiency metric (F) is calculated as:

(15) $$F=\frac{1}{2}\left(\frac{N_{t}^{\mathrm{target}}}{N_{\mathrm{all}}^{\mathrm{target}}}+\frac{N_{b}^{\mathrm{nontarget}}}{N_{\mathrm{all}}^{\mathrm{target}}}\right)\times 100\%$$

where exit t≠b is the designated exit for the target particles, $N_{t}^{\mathrm{target}}$ denotes the number of target particles exiting from the top exit, i.e., exit t. A value of 100 % means all the target particles are going through the top exit and all the nontarget particles are going through the bottom exit. For the harvesting model, the target particles were microalgal cells with larger than a chosen diameter or a constant diameter with a given composition, whereas for the fractionation model the target particles were the cell components to be isolated after an assumed disruption step. The cell and component parameters used in simulations are shown in (Table 2).

**Table 2:** **Parameters used for T-iso cells and their components.** Subscripts _SC_, _CO_, _LT_, _ND_ refer to the starting culture, control, low-temperature stress and nitrogen deprivation groups, respectively. “d” is the diameter, “ρ” is the specific gravity and “c” is the speed of sound. The subscripts _cell_, _CH_, _LI_, _NU_ and _ST_ refer to the microalgal cells, chloroplasts, lipid droplets, nuclei and starch granules respectively. n = 3.

Parameter(unit)	Mean	Standarddeviation	Reference
$d_{cell}\left(\mu m\right)$	6	0.66	Thisstudy
$\rho_{SC}\left(kgm^{\left(-3\right)}\right)$	1055.7	1.91	Thisstudy
$\rho_{CO}\left(kgm^{\left(-3\right)}\right)$	1048.7	0.73	Thisstudy
$\rho_{LT}\left(kgm^{\left(-3\right)}\right)$	1059.3	0.18	Thisstudy
$\rho_{ND}\left(kgm^{\left(-3\right)}\right)$	1065.0	0.87	Thisstudy
$c_{SC}\left(ms^{\left(-1\right)}\right)$	1514.9	2.77	Thisstudy
$c_{CO}\left(ms^{\left(-1\right)}\right)$	1515.4	1.21	Thisstudy
$c_{LT}\left(ms^{\left(-1\right)}\right)$	1517.4	1.43	Thisstudy
$c_{ND}\left(ms^{-1}\right)$	1520.2	0.87	Thisstudy
$d_{ch}\left(\mu m\right)$	2	-	[67]
$d_{li}\left(\mu m\right)$	1.8	-	[68]
$d_{nu}\left(\mu m\right)$	0.8	-	[67]
$d_{st}\left(\mu m\right)$	0.5	-	[69]
$\rho_{ch}\left(kgm^{-3}\right)$	1051	0.79	Thisstudy
$\rho_{li}\left(kgm^{-3}\right)$	886	-	[70]
$\rho_{nu}\left(kgm^{-3}\right)$	1051	-	*Assumption*
$\rho_{st}\left(kgm^{-3}\right)$	1500	-	[71]
$c_{ch}\left(ms^{-1}\right)$	1516	1.54	Thisstudy
$c_{li}\left(ms^{-1}\right)$	1470	-	[72]
$c_{nu}\left(ms^{-1}\right)$	1503.5	-	[73]
$c_{st}\left(ms^{-1}\right)$	1765	-	[74]

For the harvesting model, only the input flow rate, the pressure at the quarter-wavelength section (s2) and the width of the perpendicular exit (w_b_) were taken as variables. Parameters that maximize the focusing in section 1 were chosen beforehand. All other parameters were kept constant (Table 3). The frequency of excitation was determined considering the aspects of manufacturing and component availability. A channel width of 300 µm is easily achievable by using common microfluidics manufacturing technologies [63]. Furthermore, the corresponding half-wave and quarter-wave frequencies of approximately 2.47 MHz and 1.235 MHz can be realized by using off-the-shelf available components from multiple manufacturers.

**Table 3:** **Parameters for the COMSOL Simulations.** Geometry and operational parameters are given in their corresponding ranges. Subscripts 1 and 2 refer to the focusing and separation sections, respectively. w_0_, w_1_, P, l, f and $\dot{V}$ refer to the width of the main channel, width of the perpendicular exit, acoustic pressure, the length, the frequency and the flow rate, respectively.

Parameter	Value
$w_{0}$	300μm
$P_{1}$	2MPa
$l_{1}$	10mm
$l_{2}$	10mm
$f_{1}$	2.47MHz
$f_{2}$	1.235MHz
$\dot{V}$	$0.5-50\;\mu L\;min^{-1}$
$w_{b}$	150-600μm
$P_{2}$	0.1-2MPa

The COMSOL® simulations were coupled to an optimization loop in MATLAB® r2022b in order to find the set of parameters that ensure the separation threshold is achieved while maximizing the efficiency metric. The coupling is realized through COMSOL’s LiveLink with MATLAB interface. The objective function is created such that it first runs the COMSOL simulation with given parameters, and returns the objective value to be minimized, which is the inverse of the efficiency metric. This arrangement eventually looks for increasing the efficiency. Following the Bayesian optimization workflow, the algorithm first determines the next set of parameters to evaluate, and this loop continues until further improvements are not possible. Bayesian optimization used as optimization algorithm for its several advantages over other methods, such as gradient-based methods and monte-carlo or genetic algorithm methods [64]. The search space of parameters are discretized so that the physical realization of the concept devices are simpler to manufacture. Bayesian optimization is also a gradient-less method, which avoids thousands of objective function evaluations. This is particularly useful as the objective function is expensive to evaluate. Thus, one simulation with 100 s of particles last in the order of minutes on an average computer. Finally, it is a global optimization algorithm able to identify the minimum or maximum over a given set, rather than their local values.

First, the set of parameters were sought for the harvesting step, with the goal to harvest cells greater than a given diameter. Then, another harvesting experiment was simulated where microalgal cells of identical size but different composition, e.g. culture conditions, were harvested. The size-based harvesting inquires the possibility of only harvesting microalgal cells that are mature, i.e. grown to a certain point, whereas smaller microalgal cells may be grown further. The composition-based harvesting inquires the feasibility of separating microalgal cells that have slightly different ACFs. Within this research, this change in composition is presumably achieved through different culture conditions, however, it is generally considered that even within the same culture, microalgal cells may differ significantly in their compositions and hence ACFs. It is expected that the differences in ACFs may be too small to reach acceptable fractionation efficiencies, hence, as an alternative, dynamic-field based separation methods were evaluated for microalgal cells with different compositions. In between the harvesting and fractionation, a disruption process is assumed, based on our previous work [17]. This allows the release in the channel of various cell structures such as intact chloroplasts, lipid droplets, starch granules and cell nuclei. Subsequently, the parameters of the fractionation model were optimized to isolate these cell components from the mixture at a high efficiency.

## 3 Results and discussions

### 3.1 Acoustic properties of intact T-iso cells and internal structures

Acoustic properties such as specific gravity and compressibility of single cells and their components will directly impact how they behave when subjected to acoustic forces. Thus, it is imperative to determine these traits in order to understand the effect of these forces on these cells as well as individual intracellular structures. To achieve this, the ACF of various T-iso cultures was determined. These were cultivated in optimal as well as stressed conditions in order to induce changes in their biomass composition and thus determine if these will in turn impact the acoustic properties of these cells. Subsequent investigations were performed on the isolated chloroplast of T-iso. Main components such as lipids, proteins, carbohydrates and pigments were measured and their impact on the cell specific gravity and compressibility discussed.

**Fig. 2:**
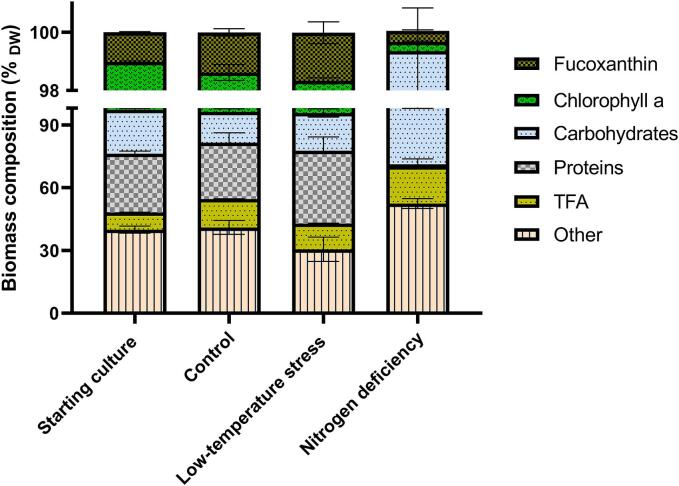
**Compositional changes in the T-iso microalgal biomass as a result of different cultivation regimes.** The content of the starting cultures is compared with the final biomass composition after 20 days of cultivation under optimal (control) as well as stressed growth conditions. Depicted are different pigments, as well as carbohydrates, proteins and TFA (Total Fatty Acid) content.

There are notable changes observed in terms of biomass composition between the starting culture and the ones cultivated under different growth conditions. The initial culture showed a typical composition, with around 28 % proteins, followed by 21 % carbohydrates, 8 % total fatty acids and 3 % pigments (Fig. 2). The T-iso culture cultivated under optimal growth conditions did not show a substantial change in this initial composition, except for a slight decrease in carbohydrates content, to 15 %, and increase in fatty acids to 13 %. However, the two stressed conditions, low temperature and nitrogen deficiency, indeed led to significant changes in biomass composition of T-iso. For instance, a considerably higher protein concentration of above 35 % was measured in the case of temperature-stressed cultures, while 17 % of total fatty acids was determined in the nitrogen-limited one. The former also exhibited slightly higher pigment content of 4–5 %, while the latter manifested a higher carbohydrate content of 28 % and very low amounts of proteins.

**Fig. 3:**
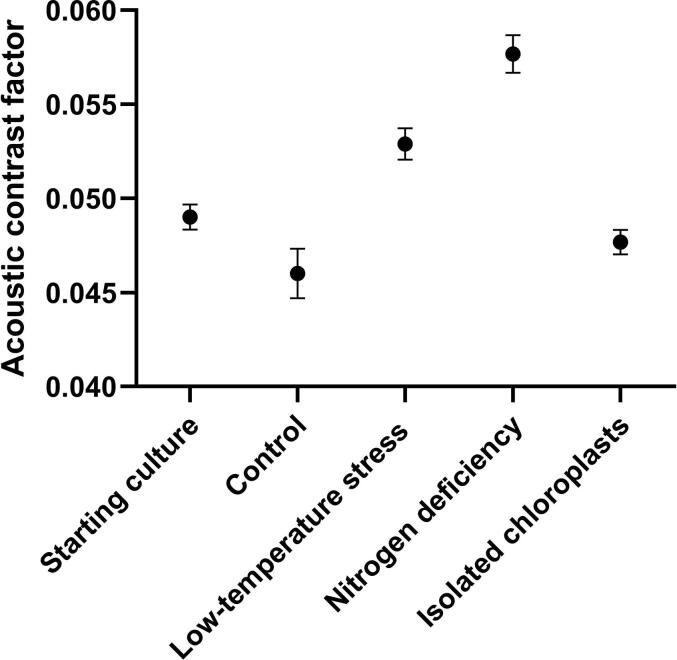
**Changes in the acoustic properties of T-iso microalgae.** The ACF is measured at the starting point of T-iso cultures as well as after 20 days of cultivation under optimal and stressed growth conditions. The chloroplasts were isolated from 20-d ay-old cultures cultivated under optimal conditions. n = 3.

Similarly, significant changes in the acoustic properties of T-iso cells were observed based on the different culture conditions. A 6 % decline from 0.049 to 0.046 was measured in the ACF when comparing the initial culture to the one cultivated under optimal conditions after 20 days (Fig. 3). On the contrary, when comparing this with the two stressed cultures, a 9.5 % increase in the ACF to 0.0527 and 0.0577 was observed in the temperature stressed and the nitrogen deprived, respectively. The ACF of the isolated chloroplasts was measured at 0.0477, situating it at a value between the starting culture and the 20 days old control culture.

The various changes observed in the biomass composition and the ACF could be connected. For instance, a slight decrease in carbohydrate content, often stored in the cell as starch granules around the pyrenoid, and slight increase in fatty acids, often accumulated by microalgae as lipid droplets, was observed in the T-iso cultures cultivated under optimal conditions. This could explain the lower ACF seen between the starting culture and this one, as starch has a positive ACF value while lipid droplets have a negative ACF value. The sum of these changes could thus reduce the overall ACF of these cells. Similar observations have been made by Gómez and collaborators, where the ACF of *Chlamydomonas reinhardtii* starch null sta6 mutant (CC-4348) cells experienced a dramatic shift from a positive to a negative ACF as the culture was stressed and accumulated more lipids and less carbohydrates [40]. This could also explain the dramatic increase in the ACF of the temperature stressed and nitrogen deficient cultures, where the protein concentration in the former and carbohydrate concentration in the latter, both with strong positive ACF, was the highest of all investigated T-iso cultures. The ACF of the isolated chloroplasts is situated slightly above the control 20 days old culture from where it was isolated. As this culture contains 13 % total lipids and the chloroplast mostly contains proteins and pigments, this could explain the slightly higher ACF of the chloroplast.

In conclusion, the biomass composition of T-iso cells directly impacts their acoustic properties, as expected. The combination of positive ACF (proteins and carbohydrates) as well as negative ACF (lipids) give these cells an overall positive contrast factor as T-iso is not known to accumulate high amounts of lipids even under stressed conditions. Moreover, most of the T-iso cells are composed of a large chloroplast, which has an ACF similar to the cells it originates from, albeit in general slightly higher as it contains less total lipids. These insights suggest the potential of using acoustic radiation forces to specifically separate these cells based on their differences in biomass composition, and even separate individual intracellular structures from disrupted biomass. However, whether these differences in size and ACF are sufficient to perform acoustophoresis-based separation requires further investigation.

### 3.2 Numerical studies on T-iso harvesting and fractionation chip

To improve the understanding of the influence of different particle sizes and compositions on separation behavior and efficiency for both microalgal cells and their cell components, numerical studies were set up and COMSOL® Multiphysics 6.1 was used to simulate the systems. The goal was to determine the parameters which result in a high separation efficiency within the common architecture and given parameter ranges. To simplify the simulations, the harvesting of the microalgae and fractionation of their cell components was simulated in separate stages, as shown in the following subchapters. In practice, these distinct stages would occur on the same chip, with a disruption chamber spatially in-between.

#### 3.2.1 T-iso harvesting based on their size and composition

The harvesting models simulated the size- and composition-based harvesting of T-iso microalgal cells and were set up using the common architecture (Fig. 1). For the size-based harvesting, the simulated particle diameters (4-8μm) were based on commonly observed T-iso size distributions and the particles were randomly assigned a composition based on the results from the experimental work in section 3.1. Thus, for each size, a relatively large distribution of ACFs was present, which in practice would simulate the worst-case scenario, i.e. a culture in which a lot of variations between the different microalgal cells are present. The specific gravity and density values for the particles were sampled from a gaussian distribution, the mean and standard deviation are taken from Table 2. For the composition-based harvesting, the simulated particles were set to the mean diameter of T-iso microalgal cells, 6 µm. In both cases, the aim was to have a clear-cut separation, i.e. high efficiency metric (F ⪆ 70 %), for a given size or composition between the top (exit t) and bottom (exit b) outlets. COMSOL® simulations, coupled to the MATLAB® optimization loop, were used to find the parameters that led to a clear-cut separation of the desired T-iso microalgae.

**Fig. 4:**
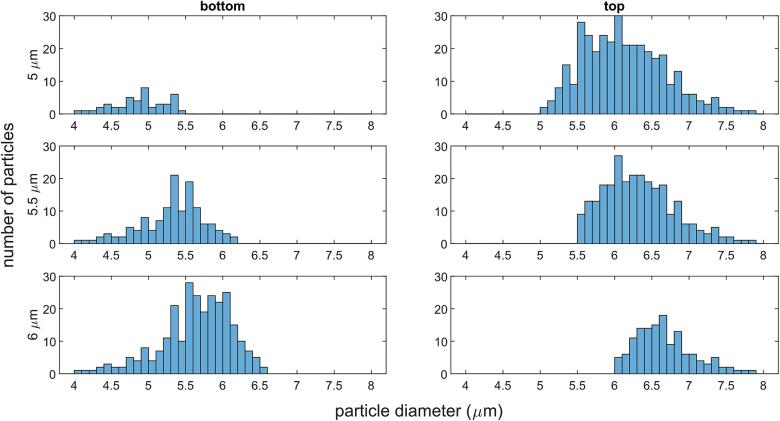
**Efficiency of T-iso cell separation using 5 µm, 5.5 µm, and 6 µm thresholds.** The same set of random particle size distributions (between 4 µm and 8 µm) was used in all scenarios for relevant comparisons. The top and bottom outlets are synonymous with the exits t and b respectively, as depicted in Fig. 1.

For the size-based harvesting, high values of the efficiency metric were found for thresholds from 5.0 µm up to 7.0 µm. For other thresholds, the efficiency metric did not reach acceptable values (F ⪅ 70 %) within the given parameter limits. Flowrates ranged from 10 µl min^−1^ to 22 µl min^−1^ and the pressure needed to be generated in section 2 was between 1.4 MPa and 2 MPa (Table 4). For thresholds of 5.0 μm, 5.5 μm, and 6.0 μm, the corresponding particle size distributions showed a clear cut-off point where only the target microalgae were harvested and no non-target microalgae were found in the top exit t (Fig. 1, Fig. 4). For the composition-based harvesting, the simulations identified that a clear-cut separation was not possible. However, when neglecting the standard deviations between samples, the optimization loop could determine the set of parameters that achieved a high separation efficiency (Table 5). In that case, the efficiency metric, as expected when neglecting the standard deviations, reached 100 %. Flowrates were also higher than what was observed for the size-based harvesting, ranging from 49 µl min^−1^ to 56 µl min^−1^.

**Table 4:** **Optimized parameters for size-based harvesting of microalgal cells.** High separation efficiency was achieved between the 5 µm and 7 µm thresholds within the given parameters.

Threshold $\left(\mu m\right)$	$\dot{V}\left(\mu l\;{min}^{-1}\right)$	$P_{2}\left(MPa\right)$	$w_{b}\left(\mu m\right)$	$F\left(-\right)$
5.0	14	1.978	510	97.98%
5.5	11	1.794	600	91.98%
6.0	13	1.770	600	84.07 %
6.5	10	1.426	600	79.78 %
7.0	22	1.943	600	69.20 %

**Table 5:** **Optimized parameters for composition-based harvesting of microalgal cells.** For all culture conditions (ND = Nitrogen deprived, LT = Low-Temperature stress, SC = Starting Culture) the cell diameter is set to 6 µm and cell parameters are taken from Table 1, ignoring standard deviations, and resulting in an efficiency metric of 100 %.

Top Outlet	Bottom Outlet	$\dot{V}\left(\mu l\;{min}^{-1}\right)$	$P_{2}\left(MPa\right)$	$w_{b}\left(\mu m\right)$	$F\left(-\right)$
ND	CO SC LT	56	2000	600	100 %
ND LT	CO SC	53	2000	600	100 %
ND LT SC	CO	49	2000	600	100 %

**Fig. 5:**
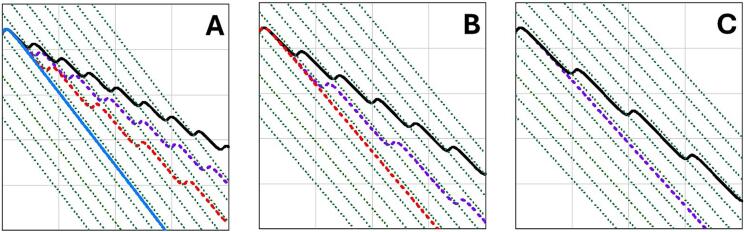
**Trajectories of T-iso microalgae in a two-frequency dynamic acoustic fields.** The starting culture (SC) is represented in purple, while the control (CO) in black, cultures cultivated under low temperatures (LT) in red and the ones under nitrogen deprivation (ND) in blue. The average employed frequency is 2 MHz, and the frequency difference in (a) is 3.5 Hz where only ND is captured, in (b) 3.2 Hz and only LT and ND are captured and in (c) 3 Hz and SC, LT, and ND are captured. Green lines depict the trajectories of pressure nodes, with a distance of 0.37 µm between each node. Timespan in all cases is 4 s.

When it came to harvesting microalgal cells larger than a certain diameter, a clear-cut separation could be achieved at the top outlet for certain diameters (5.0 µm, 5.5 µm, 6.0 µm) (Fig. 4). At the bottom outlet, however, there were target cells exiting and this reduced the harvesting efficiency. This is linked to the range of specific gravities and speeds of sound (Table 2), therefore it is possible to have a larger cell experiencing smaller acoustic force than a smaller cell. Nevertheless, these recirculated target cells would be further grown and rerun through the harvesting chamber and eventually harvested. When it comes to harvesting microalgae of a given composition, it was possible to achieve composition-based harvesting in significantly higher flow rates and with 100 % efficiency. However, it is possible that the larger variation in specific gravity and compressibility values of individual cells would reduce the real-world efficiency of such a system, potentially requiring smaller flow rates. This is due to the larger standard deviations observed between the measured samples, and hence a smaller difference in average ACFs. Thus, the differences observed in terms of biomass composition were offset by larger variations in the ACF within microalgae grown in the same culture conditions. This is to be expected as a culture contains a heterogenic population of cells, with slight variations in their content, even if overall these cells are synchronized to a certain extent through careful control of their cultivation conditions. A potential improvement in the design of this stimulation study would be to use moving stationary fields, i.e. dynamic acoustic fields. These can be utilized in multi-wavelength resonators. With dynamic acoustic fields it is easier to reach the exact value for the ARF needed to separate components, as the force experienced by the particles is dependent on the frequency difference between the two sound sources. This frequency difference is more tunable and controllable than the pressure amplitude profile within a channel and can hence lead to a more stable and adaptable separation process. To illustrate this, a case with two opposing transducers operating at an average frequency of 2 MHz and a total peak acoustic pressure of 2 MPa has been set up numerically to demonstrate the separation of T-iso microalgae of different culture conditions (Fig. 5). Further details on the theory behind the dynamic acoustic fields and how the systems are realized can be found in [21], [38], [39], [65].

To conclude the size- and composition-based harvesting of T-iso microalgae, the numerical analysis has shown that separating microalgae based on size is possible at very high separation efficiencies for size thresholds 5.0 um to 7.0 um. Even though in the worst case, i.e. the microalgal particles were assigned an ACF from different culture conditions, this difference was not important enough to hinder separation significantly. On the other hand, this meant that separating solely based on composition, and hence ACF, would be difficult. These suspicions were confirmed with the composition-based harvesting model and only after neglecting the standard deviations associated with the composition measurements was it possible to separate the microalgae. To improve the separation process in the future, dynamic acoustic fields promise a higher tunability and offer more control on the forces the particles experience by changing the excitation frequency or phase offset between the two sound sources. While such fields usually require a larger resonator, with careful design considerations the effect of deviations in cell parameters may also be compensated.

#### 3.2.2 T-iso microalgal cell component fractionation

**Fig. 6:**
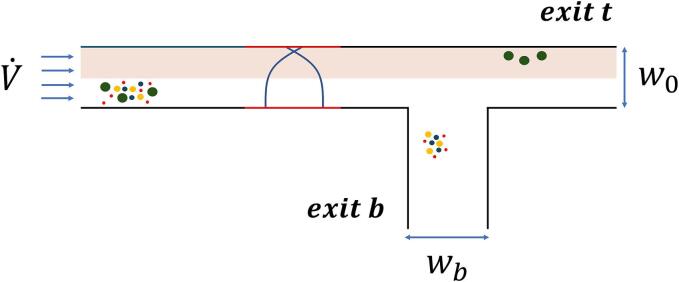
**Modified acoustic microfluidic architecture for the separation of T-iso cell components.** The main microalgal cell constituents, depicted by different colours, are entering the separation chamber from the lower half while a buffer fluid (light red) is introduced into the upper half of the channel. The quarter-wavelength acoustic standing wave separates in this case the chloroplasts from the other microalgal cell components due to its stronger interactions with the acoustic pressure node situated towards the top wall of the channel. The lipid droplets are depicted in yellow, chloroplast in green, nuclei in blue and starch granules in red.

The T-iso microalgal cell component fractionation takes place right after the presumed disruption step and simulates the separation of T-iso microalgal cell components. The fractionation model was constructed with a repeating pattern of a variation of the common architecture (Fig. 6). The goal was to separate the first cell component from the cell component mixture in the first instance of the common architecture, then the second cell component from the mixture in the second instance of the common architecture, et cetera. However, the microalgal cell components were considerably smaller than microalgal cells, thus it was necessary to increase the time of flight to facilitate the separation process. To achieve this, the cell components entered the channel from the bottom half of the channel and no focusing section (s1) was required. Buffer liquid was provided for the top half of the channel. In the case of separating lipid droplets, the buffer inlet and cell component inlets were inverted as the lipid droplets have a negative ACF, hence experience an acoustic radiation force in the opposite direction. First the lipid droplets components are separated, exiting through exit b. In the following two stages, first the chloroplasts and then the nuclei are exiting through exit t in consecutive steps, leaving only starch components exiting from the bottom (exit b) and thereby concluding the fractionation. The average diameters and densities of chloroplasts, lipid droplets, nuclei, and starch granules were retrieved from experiments and literature (Table 2). The efficiency metric was determined for each component, and the optimized parameters were recorded.

The microalgal cell component fractionation simulations successfully determined the optimized parameters and reached efficiency metrics of 100 % (Table 6). For all the cell components, the peak acoustic pressure was equal to the maximum value, 2000 kPa. The flow rates were considerably lower, close to a factor of 10 compared to the cell harvesting and ranged from 0.9 µL min^−1^ to 3.0 µL min^−1^. The fractionation process assumes no variation in size and ACF within each specific category of microalgal cell components, which explains the perfect separation efficiency as well as the fixed value for the peak acoustic pressure. Due to the smaller size of the cell components, they experience a considerably lower acoustic radiation force and the process was slower than the microalgae harvesting. A remedy is to operate the system in the appropriately higher acoustic pressure, while another option is to scale down the channel and use higher frequencies. However, these conditions could lead to acoustic streaming phenomena, hindering the process [28]. As proposed in section 3.2.1, dynamic acoustic fields could also be considered as an alternative to reach higher throughput and tunability, especially when variations in size and ACF must be taken into account.

**Table 6:** **Separation conditions for the different T-iso cell components.** For each stage, the peak acoustic pressure is 2000 kPa and the flow rate indicates the total flow rate, including the buffer liquid coming from either the top or the bottom (for lipid droplets) half of the channel. The efficiency metric reached 100 % for all cell components.

Microalgal cell component	$\dot{V}\left(\mu l\;{min}^{-1}\right)$	$w_{b}\left(\mu m\right)$
Lipid droplets	3.0	300
Chloroplast	2.0	600
Nucleus	0.9	450

The fractionation of different types and sizes of microalgal cell components was demonstrated in a numerical study. The size and acoustic properties were different enough between the various microalgal cell components to reach clear-cut separation within the simplified model. To further simplify the models and substantially accelerate the optimization process, the acoustic streaming forces were not considered within the models. However, though they play a negligible role for larger particles such as the microalgal cells, previous research has clearly demonstrated that the movement of sub-micron particles, such as the starch granules and nuclei, may be predominately determined by acoustic streaming forces. These are dominating over the acoustic radiation force in these instances [66]. Hence, to build on these results, an extended model that incorporates acoustic streaming forces coupled to experimental results is necessary.

## 4 Conclusion

As a first step towards a multiproduct biorefinery on a chip, the microalgal cell composition was analyzed under different culture conditions and related to their acoustic properties. The specific gravity and compressibility of microalgal cells and main structures such as the chloroplast were indirectly determined using their neutrally buoyant state. The ACF ranged from 0.046 for the optimal growth culture up to 0.0577 for the nitrogen deprived culture. The numerical study suggested that size-based harvesting, along with the subsequent separation of cell components, are feasible using the data obtained in this research and relevant components from literature. Composition-based harvesting proved difficult due to the minimal differences in ACF between the T-iso microalgae in different culture conditions and hence separation was only possible when neglecting the standard deviations of the ACFs. Apart from composition-based harvesting, these findings indicate that the differences in acoustic parameters of the different T-iso cells and cell components are sufficient to envision a label-free multiproduct biorefinery on a chip, using just acoustic forces. To the best of our knowledge, this is the first time this approach has been investigated. Further research should however focus on experimentally demonstrating these *in silico* findings using different microalgal cells, as well as integrating the different processing steps such as cell harvesting and disruption as well as product fractionation. Moreover, the scaling-out potential of this novel approach through process parallelization should be explored. The present investigation has laid the groundwork for further exploration of the herein described acoustofluidic processing of microalgae as a means to alleviate the downsides of existing downstream processing steps and hence expedite the shift toward a more sustainable, biobased economy.

## CRediT authorship contribution statement

**Jacques R.N. Kieffer:** Writing – original draft, Visualization, Software, Methodology, Investigation, Formal analysis, Data curation, Conceptualization. **Hakan Kandemir:** Writing – original draft, Visualization, Software, Methodology, Investigation, Formal analysis, Conceptualization. **Lars Stegemüller:** Writing – review & editing, Visualization, Methodology, Investigation, Formal analysis. **Isa Hiemstra:** Writing – review & editing, Visualization, Methodology, Investigation, Formal analysis. **Michel H.M. Eppink:** Writing – review & editing, Supervision, Methodology. **Rene H. Wijffels:** Writing – review & editing, Supervision. **Iulian Z. Boboescu:** Conceptualization, Data curation, Formal analysis, Funding acquisition, Investigation, Methodology, Project administration, Resources, Visualization, Supervision, Writing – review & editing.

## Funding

This project has received funding from the European Union’s Horizon 2020 research and innovation programme under the Marie Sklodowska-Curie grant agreement No 845185, and the NWO Science XS programme, under project number OCENW.XS5.043.

## Declaration of competing interest

The authors declare the following financial interests/personal relationships which may be considered as potential competing interests: [Iulian Zoltan Boboescu reports financial support was provided by Marie Sklodowska-Curie. Iulian Zoltan Boboescu reports financial support was provided by NWO Science XS. If there are other authors, they declare that they have no known competing financial interests or personal relationships that could have appeared to influence the work reported in this paper].

## Figures and Tables

**Supplementary Figure 1:**
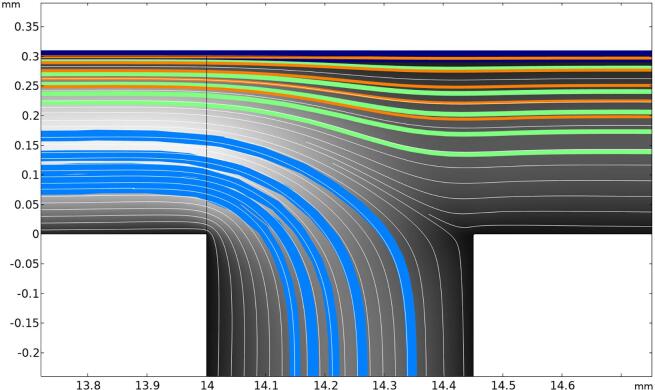
Simulated velocity fields, streamlines and trajectories of different cell components during the separation of lipid droplets. The velocity field is represented in grayscale in relative scale, with brighter areas indicating faster flow velocity while dark areas slower velocities. The velocity streamlines are depicted in greylines and the various particle trajectories in colored lines. The lipid droplets are depicted in light blue, chloroplasts in dark blue, nuclei in green and starch granules in orange lines. Line thickness is proportional to particle radii. The number of particles is reduced to improve visibility. The acoustic pressure field is considered as background pressure field.

**Supplementary Figure 2:**
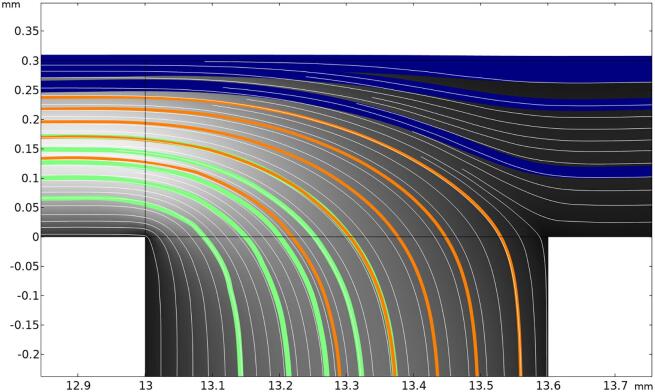
Simulated velocity fields, streamlines and trajectories of different cell components during the separation of chloroplasts. The velocity field is represented in grayscale in relative scale, with brighter areas indicating faster flow velocity while dark areas slower velocities. The velocity streamlines are depicted in greylines and the various particle trajectories in colored lines. The chloroplasts are depicted in dark blue, nuclei in green and starch granules in orange lines. Line thickness is proportional to particle radii. The number of particles is reduced to improve visibility. The acoustic pressure field is considered as background pressure field.

**Supplementary Figure 3:**
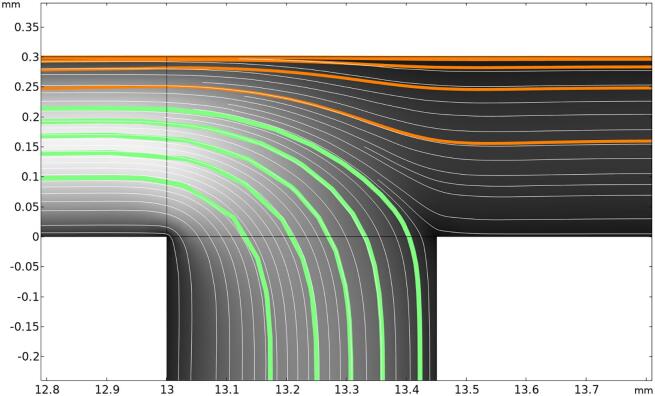
Simulated velocity fields, streamlines and trajectories of different cell components during the separation of nuclei from starch granules. The velocity field is represented in grayscale in relative scale, with brighter areas indicating faster flow velocity while dark areas slower velocities. The velocity streamlines are depicted in greylines and the various particle trajectories in colored lines. The nuclei are depicted in green and starch granules in orange lines. Line thickness is proportional to particle radii. The number of particles is reduced to improve visibility. The acoustic pressure field is considered as background pressure field.
